# Vom Sinn des Sinns als geteiltem Sinn – über Führungsentwicklung in turbulenten Zeiten

**DOI:** 10.1007/s11612-020-00540-y

**Published:** 2020-10-27

**Authors:** Andrea Kleinhuber, Anja Hermann

**Affiliations:** 1 Personal- und Führungsentwicklung, Universitätsspital Basel, Klingelbergstr. 23, 4031 Basel, Schweiz; 2 Bereichsleitung Pflege Medizin, Universitätsspital Basel, Hebelstr. 2, 4031 Basel, Schweiz

**Keywords:** Führungsentwicklung, Shared Leadership, Führungskultur, Sinngemeinschaften, Veränderungsprozesse, Organisationales Lernen, Leadership development, Shared leadership, Leadership culture, Communities of Meaning, Change, Organizational learning

## Abstract

„In der Krise beweist sich der Charakter“ (Helmut Schmidt) – und die Kultur einer Organisation. Trägt sie auch in schwierigen Zeiten? Kommt es unter Druck zu einer Verstärkung der positiven oder der dysfunktionalen Aspekte? Dieser Praxisbericht befasst sich mit einem Pflegebereich eines Universitätsklinikums und insbesondere seiner Führungskultur, die jüngst angesichts der COVID-19-Pandemie einem „Stresstest“ ausgesetzt war. Erleichternderweise trat hier ein solides Fundament zutage bzw. es zeigte sich die positive Kraft einer bereichsumfassenden Kultur der gemeinschaftlichen Führung. Unser Beitrag zu diesem Themenheft der Zeitschrift Gruppe. Interaktion. Organisation. (GIO) reflektiert einen fünfjährigen Führungsentwicklungsprozess, der die Herausbildung einer adaptiven und gemeinschaftsorientierten Führungspraxis, welche Komplexität, permanente Veränderung und multiple Spannungsfelder konstruktiv bewältigen kann, unterstützt hat. Kernelemente dieses Prozesses waren regelmäßige Großgruppenworkshops, die als Resonanz‑, Reflexions- und Experimentierräume dienten sowie Vertrauen und Verbundenheit förderten. Wir rekapitulieren zunächst wesentliche Schritte und Inhalte des Prozesses und beleuchten dann auf Basis von Interviews mit beteiligten Führungspersonen Wirkfaktoren und Gelingensvoraussetzungen der stattgefundenen Entwicklungen. Anschließend erfolgt eine Auseinandersetzung mit theoretischen Perspektiven auf Sinnfragen in Zusammenhang mit Führung und eine Einordnung und Diskussion des Fallbeispiels vor diesem Hintergrund. Dabei interessieren uns vor allem solche Ansätze, die Sinnphänomene als transsubjektiv und kollektiv betrachten und unser Beitrag plädiert dafür, Sinn nicht losgelöst von den Gemeinschaften zu denken, in welchen er entsteht. Wir enden mit einem Resümee und Ausblick angesichts einer Reorganisation im Kontext des Fallbeispiels.

## Abstract (en)

“In a crisis character proves itself” (Helmut Schmidt)—and so does the culture of an organization. Is it of support also in difficult times? Does pressure amplify its positive aspects or rather the dysfunctional ones? This case report addresses a nursing division within a university hospital, particularly its leadership culture that was recently “stress tested” by the COVID-19 pandemic. To the relief of those involved, the situation revealed a solid foundation and the positive force of a division-wide culture of shared leadership. Our contribution to this special issue of the journal Gruppe. Interaktion. Organisation. (GIO) reflects upon a leadership development process of five years, which supported the emergence of an adaptive and community-oriented leadership practice capable of constructively coping with complexity, permanent change and various tensions. Core elements of this process were regular large group workshops that provided spaces for resonance, reflection and experimentation and fostered the building of trust and connectedness. We first recapitulate significant steps and contents of this process and then examine impact factors and determinants of success by drawing on interviews with participating leaders. This is followed by an engagement with theoretical perspectives on questions of meaning in relation to leadership and a consideration and discussion of the case example against this background. In that we focus on such approaches that look at meaning phenomena as transsubjective and collective and our article advocates against considering meaning detached from the communities in which it emerges. We conclude with a résumé and outlook in view of a reorganization in the context of this case study.

## Einleitung

In der Welt der Unternehmen ist jüngst mit dem „Purpose“-Begriff die Frage nach dem übergeordneten Sinn oder dem höheren Existenzgrund jenseits des finanziellen Gewinns in den Fokus gerückt. Ob es sich dabei um eine unternehmerische Überlebensfrage oder einen unnötigen Hype, um ehrenwerte oder aufgesetzte moralische Selbstfindung oder schlicht um zeitgeistsensibles Marketing handelt, daran scheiden sich die Geister in einschlägigen Internetforen und Fachmedien. Weitgehende Einigkeit scheint jedoch darüber zu bestehen, dass insbesondere die jüngeren Generationen Y und Z vermehrt von dem Wunsch geleitet sind, mit ihrer Arbeit zu etwas Sinnhaftem beizutragen. Organisationen des Gesundheitswesens können zwar im Branchenvergleich nicht mit Topgehältern oder den komfortabelsten Arbeitsbedingungen punkten, bieten dafür aber berufliche Einsatzmöglichkeiten mit hohem Sinnbezug und offensichtlicher gesellschaftlicher Relevanz. Angehörige des Pflegeberufs weisen generationenübergreifend eine überdurchschnittliche Identifikation mit ihrer Arbeit auf und ihr Sinnerleben im Einsatz für Gesundheit und Wohlbefinden von Patientinnen und Patienten wirkt als Ressource für die Bewältigung einer häufig hohen Arbeitsintensität mit physischen und psychischen Belastungen. Gleichzeitig bietet Sinnhaftigkeit allein noch keinen Schutz vor Gesundheitsrisiken bei der Arbeit, wie hohe Burnout-Raten bei den helfenden Berufen zeigen (Schmucker 2020). Auch besteht die Gefahr des Sinnverlustes, wenn die Arbeitsbedingungen die Erfüllung verinnerlichter Wertvorstellungen und Qualitätsmaßstäbe verhindern oder im Zuge der Ökonomisierung des Gesundheitswesens Effizienz und Gewinn als über dem Patientenwohl und der Mitarbeitendengesundheit stehend erlebt werden (Hinding und Kastner 2013).

Führungspersonen in der Pflege sehen sich vor der Herausforderung, das Spannungsverhältnis zwischen wachsendem wirtschaftlichen Druck auf der einen sowie Qualität und Sicherheit auf der anderen Seite gemeinsam mit ihren Teams konstruktiv zu gestalten und dabei die Sinnpotenziale des Pflegeberufs zu bewahren. Gleichzeitig befindet sich das Gesundheitswesen im Umbruch und vielerorts müssen umfassende organisationale Veränderungen bewältigt werden. Die folgende Falldarstellung befasst sich mit einem Bereich des Universitätsspitals Basel (USB), welches in dieser Hinsicht keine Ausnahme darstellt. Seit 2012 in der Schweiz das diagnosebasierte Fallpauschalen-System eingeführt und gleichzeitig das USB zu einer selbständigen (vom Träger-Kanton unabhängigen), öffentlich-rechtlichen Institution wurde, ist der Arbeits- und Führungsalltag geprägt von hoher Dynamik in vielfältigen Veränderungsprozessen. Im Bereich Medizin galt es über 700 Pflegende auf den neuen Zukunftsweg mitzunehmen, gemeinsam den erhöhten Druck in den Arbeitsabläufen anzuerkennen und den Umgang damit zu erlernen. Die Vorbereitung einer kantonsübergreifenden Spitalfusion sorgte für zusätzliche Herausforderungen neben fortlaufenden strukturellen Änderungen und der Einführung neuer Prozesse. Angesichts der ständig neuen Frage- und Problemstellungen hätte es leicht zu Frust- und Demotivationsspiralen kommen können und die Führungspersonen im Bereich waren in diesem anspruchsvollen Kontext kontinuierlich gefordert, ihre Teams zu stabilisieren und dabei gleichzeitig weiterzuentwickeln und zur Bewältigung der Veränderungsdynamik zu befähigen.

Im Frühjahr 2020 fügte die COVID-19-Pandemie dieser Situation eine weitere, bisher unbekannte Dimension hinzu. Das USB musste nun neben Grundversorgung und universitärer Spitzenmedizin die regionale Pandemieversorgung mit entsprechender Anzahl Intensivbetten und stationärer Betreuung der schwerstkranken Patientinnen und Patienten sicherstellen – ein organisatorischer Spagat inmitten unzähliger Anforderungen. Besonders wichtig war es auch in dieser Situation, die Motivation und Arbeitsfähigkeit der Mitarbeitenden aufrecht zu erhalten, damit sie selbst in Zeiten größter Unsicherheit und organisationalem „Segeln auf Sicht“ die sicherste und beste Leistung für die zu versorgenden Patientinnen und Patienten erbringen konnten.

Um den pflegerischen Führungspersonen angesichts dieser großen Herausforderung Unterstützung zu bieten, luden die Bereichsleiterin und der Bereichsfachverantwortliche der Pflege Medizin Ende März 2020 zu Kurzworkshops mit dem Titel „Gemeinsam Führen in besonderen Zeiten“ ein. Trotz der relativ kurzfristigen Ankündigung wurde dieses Angebot rege wahrgenommen. Die Workshops boten Gelegenheit für stationenübergreifenden Erfahrungsaustausch und eine Auseinandersetzung mit veränderten und neuen Führungsanforderungen in der Pandemiesituation. Daneben standen auch die Fragen „Auf was können wir bauen, worauf können wir zurückgreifen?“ und „Wie können wir einander unterstützen?“ im Fokus. Die Stimmung in den Workshops war unerwartet ruhig und überlegt, trotz der angespannten Situation bestimmte Offenheit und gegenseitige Zugewandtheit das Klima. Die Teilnehmenden sprachen unverblümt über Belastungen, aber auch über positive Erlebnisse. Die Gelegenheit zum Austausch und zur gemeinsamen Reflexion wurde als wertvoll und wichtig befunden, gleichzeitig zeigten die Rückmeldungen, dass wenig Bedarf für außerplanmäßige Unterstützungsangebote gesehen wurde. Vielmehr wurde Zuversicht geäußert, dass die guten Beziehungen und die geteilte Führungskultur, die u. a. im Rahmen von regelmäßigen Führungsworkshops aufgebaut und gepflegt worden waren, eine solide Basis für die gemeinschaftliche Bewältigung der Situation darstellten.

Diese Rückmeldungen waren für die Bereichsleiterin, den Bereichsfachverantwortlichen und die begleitende interne Beraterin gleichermaßen erfreulich wie überraschend (zumindest in dieser Ausprägung). Sie regten dazu an, eine neue Perspektive auf einen vorhergegangenen fünfjährigen Führungsentwicklungsprozess zu werfen. Dieser hatte nicht zum Ziel gehabt, auf eine Pandemiesituation vorzubereiten – und doch war offenbar ein entsprechendes Fundament entstanden. Wie war das geschehen? Und wie sollte nun an das Erreichte angeschlossen und darauf aufgebaut werden?

Im Folgenden rekapitulieren wir zentrale Aspekte und Inhalte dieses Entwicklungsprozesses und untersuchen dessen Wirkungen auf der Basis von Interviews mit beteiligten Führungspersonen. Darauf folgt eine theoretische Einordnung und Diskussion mit Fokus auf Sinnaspekte und Sinnpotenziale in Verbindung mit gemeinschaftlicher Führung. Vor diesem Hintergrund erscheint die Resilienz der Führungsverantwortlichen in der Pandemiesituation als zusammenhängend mit der Qualität der Entwicklungs- und Sinngemeinschaft, die über die Jahre im Bereich Medizin gewachsen ist. Entsprechend plädiert unser Beitrag dafür, Sinn nicht isoliert von relevanten Beziehungsnetzwerken und den durch sie generierten Möglichkeiten der Bewältigung von Sinnanfragen zu betrachten. Die Reflexion des Fallbeispiels endet mit einem Resümee und Ausblick im Kontext einer anstehenden Reorganisation des betreffenden Organisationsbereichs.

## Die Führungsentwicklungsreise der Jahre 2015–2019

Anfang 2015 beauftragten die Leiterin und der Fachverantwortliche der Pflege Medizin^1^ die neueingetretene Leiterin Führungsentwicklung des USB mit der Konzeption und Moderation von Führungsentwicklungsworkshops im Bereich. Diese Zusammenarbeit verstetigte sich in der Folge und fand ihren Ausdruck nicht nur, aber schwerpunktmäßig in regelmäßig stattfindenden Workshops (drei Tage pro Jahr) mit dem gesamten Pflegekader. Hierzu zählen im Bereich Pflege Medizin Stationsleitungen und ihre Stellvertretungen sowie Fachverantwortliche und Hauptverantwortliche Berufsbildner. Von den etwa 60 Kaderpersonen konnten durchschnittlich ca. 45 pro Workshop teilnehmen, es handelte sich also um Großgruppenveranstaltungen.

Das Vorgehen war von Beginn an ein dialogisches, in welchem bottom-up Impulse und top-down Zielsetzungen miteinander verflochten wurden. Im Rahmen einer Fokusgruppe mit Angehörigen des Pflegekaders waren deren Anliegen und Schwerpunktthemen erhoben worden. Diese galt es zu verbinden mit dem Ziel der Bereichsleitung, das Thema „Shared Governance“ bzw. die gemeinschaftliche Führung im Bereich zu stärken und auszuweiten. Nachdem die sog. „duale Führung“ von Linie und Fach bereits etabliert worden war, sollten nun zusätzlich die Bildungsverantwortlichen in den Kaderkreis aufgenommen werden und die bisherigen Führungstandems sich zu erweiterten Führungsteams entwickeln.^2^

Die Workshops des ersten Jahres befassten sich mit eher grundlegenden, aufeinander aufbauenden Themen: 1) den individuellen Führungsrollen, 2) der gemeinschaftlichen Führung in der „Führungskoalition“ und 3) der Teamführung bezogen auf die Abteilungsteams. Hierbei kam es zu wichtigen Rollen- und Werteklärungen sowie zu zentralen Auseinandersetzungen mit Chancen, Herausforderungen und Entwicklungsfeldern der gemeinschaftlichen Führung. Im Folgejahr wurde die Führungszusammenarbeit auf Bereichsebene zum Fokusthema. Dabei wurde einerseits die Zusammenarbeit mit den ärztlichen Partnerinnen und Partnern reflektiert und andererseits die stationsübergreifende Kooperation in der Pflege. Um diese Kooperation nicht nur zu thematisieren, sondern auch zu praktizieren, waren die Teilnehmenden in Varianten des Großgruppenformats Open Space dazu eingeladen, selbstgesteuert an geteilten Problemstellungen und Zielen zu arbeiten und in Kollaborationen jenseits von Abteilungsgrenzen Lösungen zu entwickeln. Daraus entstanden Initiativen, die über die Workshops hinaus gemeinsam weiterverfolgt wurden.

Im Jahr 2017 spitzte sich der Veränderungsdruck durch aktuelle Struktur- und Prozessveränderungen sowie anstehende Entwicklungen (v. a. die damals noch erwartete Spitalfusion) weiter zu. Gleichzeitig wurde zunehmend deutlich, dass eine mit derart intensiver Veränderungsdynamik konfrontierte Führung nicht auf kleinteiliges Orchestrieren setzen kann, sondern auf die Selbststeuerungsfähigkeiten ihrer Teams angewiesen ist. So rückte in diesem Jahr das Thema „Unsere Führungskultur als Fundament angesichts aktueller & zukünftiger Herausforderungen“ in den Fokus. Ein erster Workshop zielte auf die kritische Würdigung der gelebten Führungskultur im Bereich sowie auf ein reflektiertes gemeinsames Führungsverständnis ab. Im Anschluss ermöglichte ein zweiter Workshop Blicke über den Tellerrand und hinter die Kulissen anderer Organisationen, welche Selbstorganisation zum zentralen Element ihrer Organisationskultur gemacht haben.

2018 befanden wir uns vermeintlich im Endspurt auf die Fusion hin. Vor diesem Hintergrund setzten wir uns in einem zweitägigen Planspiel mit einer komplexen Change-Simulation auseinander und überprüften mentale Modelle und Vorgehensideen im Hinblick auf unhinterfragte Vorannahmen und alternative Handlungsoptionen. Ein darauffolgender Workshop zum Thema „Resiliente Führung unter Druck“ bot Raum für die Reflexion und Erweiterung eigener Coping-Strategien.

Der Beginn des Jahres 2019 schließlich war geprägt vom Schock des Fusionsstopps als Resultat einer Volksabstimmung. Die kollektive Investition in dieses Großprojekt war über Nacht hinfällig geworden. Gleichzeitig war davon auszugehen, dass das USB dennoch bald grundlegende organisatorische Veränderungen vornehmen würde. In diesem „Zwischenjahr“ rückte das Themenfeld „Lernen, Experimentieren, Potenzialentfaltung“ in den Vordergrund. Wir setzten uns mit dem Bereich als lernender Organisation auseinander sowie mit dem Thema Diversität und kollektive Kreativität. In den Workshops der vergangenen Jahre hatten wir immer wieder methodische Experimente gewagt. In diesem Jahr bildete die spontane Kreation und professionelle Aufnahme eines Songs zum Thema Lernen mit ca. 45 Personen in einem mobilen Tonstudio den vorläufigen Höhepunkt der gemeinschaftlichen Experimentierfreude und ermöglichte ein sinnliches Gemeinschaftserlebnis sowie eine Sinnerfahrung der besonderen Art.

Der nächste Workshop war für April 2020 geplant gewesen, musste aber aufgrund der Gruppengröße in der Pandemiesituation abgesagt werden. Wir hatten vorgehabt, beim Thema Lernen anzuknüpfen und uns mit generativen Ansätzen der Organisationsentwicklung zu beschäftigen.

Zusammenfassend lässt sich festhalten, dass wir in diesen fünf Jahren keinen Masterplan umgesetzt, sondern uns iterativ vorwärtsbewegt haben. Dabei sind wir durchaus einem inhaltlichen roten Faden gefolgt, den wir jedoch von Jahr zu Jahr weitergesponnen haben – im kontinuierlichen Dialog zwischen den Auftraggebern und der Prozessbegleiterin sowie in Resonanz zu aktuellen Themen und Entwicklungen und zu Rückmeldungen aus dem Führungskreis.

## Rückblickende Auswertung der Führungsworkshops und ihrer Effekte

Sicherlich ist die demonstrierte individuelle und kollektive Resilienz des Pflegekaders angesichts der Pandemiesituation nicht allein auf die Erfahrungen in den Führungsworkshops zurückzuführen. Diese sind als Puzzlestücke in einem Gesamtbild mit vielen weiteren Faktoren zu betrachten. Nachdem jedoch wiederholt auf die Workshops als Ursprungsort wichtiger persönlicher Ressourcen und eines gemeinsamen Fundaments für die Bewältigung kollektiver Herausforderungen verwiesen wurde, erschien es uns vielversprechend, diesen Hinweisen nachzugehen. Da wir bei der Planung und Gestaltung der Workshops prozessorientiert vorgegangen waren und nicht gezielt auf einen kumulativen Effekt aus waren, schien uns dieser offenbar nicht voll bewusst zu sein. Was hatte sich da in der Summe über die Jahre entwickelt? Und wodurch war das geschehen? Diese Fragen wurden für uns nicht nur im Sinne des rückblickenden Verstehens bedeutsam, sondern auch mit Blick nach vorne: Im Juni 2020 wurde am USB eine umfassende, 2021 in Effekt tretende Reorganisation angekündigt und den Bereich Medizin wird es in der jetzigen Form zukünftig nicht mehr geben. Soweit es die Entwicklung der COVID-19-Pandemie zulässt, werden wir in diesem Jahr wohl noch einen letzten Führungsworkshop in der aktuellen Konstellation durchführen können. Von einer Reflexion der vergangenen Workshops erhofften wir uns also auch wichtige Impulse für die Gestaltung dieser letzten gemeinsamen Veranstaltung sowie Inputs für Überlegungen zum „Wie weiter?“ in den neuen Konstellationen.

Um uns nicht allein auf eigene Beobachtungen und Interpretationen zu verlassen, entschlossen wir uns, Führungspersonen aus dem Bereich zu ihrer Wahrnehmung und Einschätzung zu befragen. Dabei begannen wir mit acht explorativen, semistrukturierten Interviews. Auf Basis der darin erhobenen Daten erwägen wir aktuell die Durchführung einer breiter angelegten und vertieften qualitativen Untersuchung zu den Effekten und Wirkfaktoren der Führungsentwicklung im Bereich Medizin. Vorerst stehen uns nur die Rückmeldungen aus der Voruntersuchung zur Verfügung, doch selbst dieses begrenzte Datenmaterial kann im gegebenen Rahmen nicht umfassend dargestellt werden. Im Folgenden wollen wir jedoch einige zentrale Themen und Tendenzen zusammenfassen, die für unsere Reflexion und zukünftige Planung bedeutsam erscheinen.

### Alltagsrelevanz und Vorgehensweisen

Angesichts ihres dichten Arbeitsalltags war es für die Führungspersonen im Bereich immer wieder eine Herausforderung, sich drei Tage im Jahr für die Teilnahme an den Workshops frei zu machen. Entsprechend schätzten sie die inhaltliche Alltagsnähe: „Die Themen waren immer die, welche halt gerade dran waren, insofern war es das passende Format für den dauernden Change“. So wurden die Workshops nicht als zusätzliche zeitliche Belastung, sondern als unterstützend erlebt: „Man konnte Wissen, Ideen, Alltagshilfen sammeln und seinen Rucksack vollpacken.“ Es wurde auch geschätzt, dass regelmäßig durch externe Gäste neue, z. T. auch herausfordernde Impulse eingebracht wurden: „Gut, dass wir nicht immer nur von uns ausgegangen sind, sondern auch Akzente von außen reinkamen“; sozusagen „neue Fäden zum Weiterspinnen“. Positiv erwähnt wurde auch die Mischung unterschiedlicher Formate und diverse methodische Experimente: „Da habt ihr euch schon was getraut und uns was zugetraut, aber letztlich war es immer gut, mal die Komfortzone zu verlassen und aus dem Trauen wuchsen Vertrauen und neue Perspektiven.“ Auch zur Haltung hinter dem gewählten Vorgehen in den Workshops gab es Rückmeldungen: „Da war nie Druck, man musste nichts Bestimmtes leisten oder bestimmte Erwartungen erfüllen.“ Eine Führungsperson beschrieb die Arbeitsweisen als „erwachsenengerecht“ und betonte den Stellenwert von Freiwilligkeit und Wahlmöglichkeiten: „Es gab ein Angebot, aber keinen Zwang; schon gar nicht den, etwas Vordefiniertes liefern zu müssen.“

### Erfahrungsaustausch und gemeinschaftliche Reflexion

Als besonders profitabel beschrieben alle rückmeldenden Personen den Erfahrungsaustausch im Rahmen der Workshops. „Mich hat immer wieder beeindruckt, wie viele wir sind und wie viel in diesem großen Kreis steckt. Und ich habe persönlich sehr profitiert von den Erfahrungen, die andere schon machen durften oder mussten.“ Neben dem kollegialen Mentoring wurde auch die Transparenz im Bereich und das „Mitbekommen, was bei anderen läuft“, als sehr hilfreich erwähnt. Man konnte voneinander lernen, sich inspirieren lassen und nicht zuletzt bei als „schwierig“ empfundenen top-down Entscheidungen bot der Kreis „einen positiven Fundus an Strategien, wie man damit umgehen kann“. Auch jenseits der Bewältigung akuter Herausforderungen wurden die Workshops als Reflexionsräume für wichtig und wertvoll erachtet. „Manchmal denkt man tags zuvor, eigentlich kann ich es mir zeitlich nicht leisten. Aber dann im Workshop merkt man erst, wie dringend nötig man diesen Sprung aus dem Alltagskarussell gebraucht hat.“ In diesem Sinne wurden die Workshops als „Orte zum Sinnieren“, zum Sortieren und Neuausrichten oder für „aktiv-produktive Muße“ beschrieben. Dabei wurde betont, dass neben dem persönlichen Innehalten besonders das Reflektieren in der Gruppe die Basis dafür bot, Alltagssituationen neu einzuordnen und alternative Herangehensweisen zu entwickeln. Auch die gemeinsame, kritische Reflexion des Miteinanders und der offene Dialog darüber wurden hervorgehoben. „Es ist im Bereich ein Bewusstsein entstanden, dass man, um gemeinsame Ziele zu erreichen, sich auch gemeinsam auseinandersetzen muss.“

### Kultur, Werteorientierung und Sinnbezug in der Führungsarbeit

Aus Sicht der Teilnehmenden haben die Workshops spürbar zu einer Haltungs- und Kulturveränderung in Richtung eines „echten Miteinanders“ und „gemeinsamen Vorwärtsstrebens“ beigetragen. „Wir sind als Abteilungen keine Inseln, sondern müssen zusammen tragfähige Lösungen finden.“ Die Kultur sei geprägt von Wertschätzung, Vertrautheit und einer Atmosphäre, in der angstfrei auch Kritisches und Selbstkritisches geäußert werden kann: „Ich habe in keinem der Workshops je erlebt, dass jemand angegriffen oder runtergeputzt wurde.“ Neuzugänge wurden schnell integriert. „Es wird vermittelt, dass die Fähigkeiten eines jeden geschätzt und gefragt sind, ob alter Hase oder Neuling.“ Dass neue Führungskolleginnen und -kollegen oft schon bei ihrer ersten Workshopteilnahme tragende Rollen im großen Kreis übernahmen, erklärte sich eine der Interviewten wie folgt: „Die spüren eine positive Neugier ihnen gegenüber. Und dass man bei uns lernend unterwegs sein darf, wir sind eine lernende Gruppe.“ Die Bereichsleiterin und der Bereichsfachverantwortliche wurden als kulturprägende Vorbilder beschrieben, die großes Vertrauen in Mitarbeitende sowie die Werte gemeinsamer Führungsverantwortung vorleben. „Die beiden sind immer auf Augenhöhe unterwegs – miteinander und mit uns.“

Über die Jahre sei man als Bereichsführungsteam zusammengewachsen und habe ein geteiltes Bewusstsein dafür entwickelt, „wie wichtig Führung ist und dass man sie miteinander angehen und leben muss“. Die Workshops spielten dabei eine zentrale Rolle und dienten der Rückbesinnung: „Was ist unser höherer Sinn? Wozu und für wen machen wir das?“ Sich immer wieder gemeinsam klar zu machen, dass Führung „nicht im luftleeren Raum“ stattfindet, sondern „bezogen auf ein höheres Gut“, wurde als motivierend und sinnstiftend beschrieben. „Die Workshops sind sowas wie Sinn-Tankstellen für mich“, drückte es eine Person aus. Eine andere beschrieb das Gefühl „sinnhafter Verbundenheit, wenn ich sehe, da wird eine gemeinsame Haltung sichtbar gelebt“.

### Wirkungen im Alltag

Alle Interviewten sahen Effekte im Arbeits- und Führungsalltag, die sie mit den Workshops in Verbindung brachten. Z. B. boten diese einen Rahmen für die Entwicklung von Ressourcen für den Umgang mit hoher Belastung. „Wie können wir uns selbst und unsere Mitarbeitenden befähigen, trotz allem gut mit dem Druck umzugehen?“ Solchen Fragen konnte man gemeinsam nachgehen, Ideen und Strategien entwickeln und „aus dem Prinzip ‚geteiltes Leid ist halbes Leid‘ wieder Kraft gewinnen“. Mehrfach erwähnt wurde in diesem Zusammenhang auch die Vorbildfunktion der Bereichsleiterin, „die unerschütterlich immer wieder auf das Positive fokussiert“, was auch in den Workshops spürbar war. Vor allem aber sei die Zusammenarbeit im Bereich einfacher geworden. Man kenne sich, verstehe sich besser und entwickle gemeinsame Lösungsansätze. „Das Denken entwickelt sich weg von ‚unsere Patienten, eure Patienten‘ hin zu ‚unser aller gemeinsame Patienten‘.“ Es seien Netzwerke entstanden, die sich im Alltag weiterhelfen und gerade auch während der COVID-19-Pandemie hätten sich diese als verlässlich gezeigt: „Man hat zusammengearbeitet, sich gegenseitig unterstützt und vorhandenes Knowhow gezielt abgeholt und eingesetzt.“ Weiterhin sei man verständnisvoller und großzügiger miteinander geworden. „Auch bei Konflikten kommt es nicht zum Kommunikationsabbruch“, sondern man suche weiter nach Dialog und Klärung. Die Hürde für „situativ notwendige Beziehungsklärungen“ sei deutlich niedriger geworden. Zudem wurde beschrieben, dass die Workshops zu wichtigen „trickle-down“ Effekten geführt hätten, indem verbesserte Beziehungen zwischen Abteilungen auf Führungsebene auch konstruktiveres abteilungsübergreifendes Miteinander auf Ebene der Mitarbeitenden gefördert hätten.

### Kritische Äußerungen & Verbesserungsvorschläge

Es soll nicht unerwähnt bleiben, dass in den Interviews auch Kritikpunkte und Vorschläge für alternative Vorgehensweisen erfragt und geäußert wurden. Neben persönlichen Abneigungen und Vorlieben für spezifische Formate, Methoden und impulsgebende Gäste bezogen sich die kritischen Äußerungen auf unerschlossenes Potenzial bei der inhaltlichen Transferbegleitung sowie auf die Beteiligung anderer Berufsgruppen. Da mehrheitlich kein signifikanter Zusammenhang mit der Suchrichtung und dem Erkenntnisinteresse dieses Beitrags gegeben ist, verzichten wir in diesem Kontext auf eine ausführliche Darstellung. Einige für die Planung zukünftiger Maßnahmen relevante Punkte werden aber unter Abschn. 5 noch aufgegriffen.

## Theoretische Einordnung und Diskussion

Für die Einordnung und Analyse des beschriebenen Führungsentwicklungsprozesses und seiner Effekte kämen eine Reihe theoretischer Perspektiven in Frage.^3^ Im Kontext dieses Themenheftes beschränken wir uns auf Ansätze, die sich mit Sinnfragen in Zusammenhang mit Führung befassen. Dabei interessieren uns vor allem solche, die weniger das individuelle Sinnerleben betrachten, sondern Sinnphänomene als kollektive verstehen. Ziel der Auseinandersetzung mit diesen Ansätzen ist eine weitere Differenzierung unserer rückblickenden Auswertung. Zudem möchten wir anhand unseres Fallbeispiels einen Beitrag zur aktuellen Diskussion über organisationale Bestrebungen der Sinnstiftung leisten und hierbei die Bedeutung von fortdauernden Beziehungsnetzwerken und kollektiver Selbstwirksamkeit in den Blick rücken.

Unter dem Sammelbegriff „Plural Leadership“ beschreiben Endres und Weibler (2019) unterschiedliche Formen von Führung, die im Plural stattfinden, also mehrere Personen als Führende einbeziehen. Diese reichen von Funktionalen Doppelspitzen und Dualer Führung über Verteilte Führung in Teams mit erkennbaren Teilführerschaften bis hin zu gemeinschaftlich geteilter Führung aller in einem Team oder einer Organisation mit fluider Führungsübernahme. Plural Leadership in seiner höchsten Ausprägung verstehen sie als gemeinschaftlichen Einflussprozess, den sie „Shared Leadership“ nennen und worin die Gruppe als Ganzes zum Führungsakteur wird. Dabei geht es um etwas qualitativ anderes als die Übernahme von Führungsrollen im Team und Shared Leadership entsteht nicht auf Beschluss oder durch Zuteilung. In dieser Form von Kooperation stehen nicht einzelne Personen als Führende im Fokus, sondern es geht um „gemeinschaftlich gewollte“ Anstrengungen und Verantwortung und „das kollektive Voranschreiten im Sinne der gemeinsamen Sache“ (2019, S. 16). Shared Leadership ist ein anspruchsvoller und voraussetzungsreicher Ansatz, birgt aus Sicht der Autoren jedoch großes Potenzial speziell bei komplexen, neuartigen Problemstellungen sowie auch allgemein zur Erhöhung der Innovations- und Transformationsfähigkeit von Organisationen und der Verbesserung der erlebten Arbeitsqualität von Individuen und Teams (2019, S. 23 ff.).

In empirischen Feldstudien untersuchten Endres & Weibler die relevanten Entstehungsmechanismen von Shared Leadership: „Shared Leadership hat die Chance zu entstehen, wenn kollektive Identitäten kultiviert und in Gemeinschaften einen Raum finden, in welchem sie sinnstiftend ausgelebt werden können. Shared Leadership entfaltet sein Potenzial mittels gemeinschaftlicher Lernprozesse. Dadurch kann die kollektive Perspektivenvielfalt und Intelligenz der Vielen gebündelt sowie in gemeinschaftliches Voranschreiten transformiert werden“ (2019, S. 41). Von zentraler Bedeutung ist also ein gemeinsames Lernen, wodurch der Erfahrungsschatz der Gruppe genutzt und das Gefühl gegenseitiger Verpflichtung gestärkt wird. Ein solches Lernen entzieht sich einer einfachen Implementierungslogik und kann nicht direktiv herbeigeführt werden. Es erfolgt über Kommunikationspraktiken, in welchen Sprache nicht als „Medium der Vermittlung von Führung“ (z. B. von schon festgelegten Visionen, Strategien, Zielen etc.), sondern als „Medium der Erschaffung und Ausübung von gemeinschaftlichen Führungsprozessen“ dient (2019, S. 17).

Als höchste Stufe solcher gemeinschaftlicher Lernprozesse beschreiben Endres & Weibler den Generativen Dialog – ein Modell von Fletcher & Käufer, das ihren empirischen Befunden entspricht. Diese Form des lernintensiv-schöpferischen Dialogs geht über inhaltliche Debatten und selbstreflektierenden Dialog hinaus und entsteht mit „zunehmender Beziehungsqualität“ in Form eines „ausgeprägten Wir-Gefühls“, auf Basis dessen eine Gruppe sich selbst koordiniert und gemeinsam Neues entwickelt (2019, S. 18). Voraussetzung für eine solche Dialog-Qualität sowie für Shared Leadership im Ganzen ist eine „gemeinschaftlich motivierte wertebasierte Identität“ (2019, S. 19), die sich an der übergeordnet bedeutsamen Sache und sinnstiftenden Zielen orientiert anstatt an individualistischen Interessen. Die Gruppe bietet dann Raum dafür, die „energetisierende Wirkung von kollektiver Identität“ zu erfahren, „Sinnhaftigkeit zu produzieren und zu erleben“ sowie gemeinschaftliche Selbstwirksamkeitserwartungen zu entwickeln (2019, S. 20 f.).

Die in Abschn. 3 zusammengefassten Rückmeldungen zur Erlebnisqualität und den Effekten des Miteinanders in den Workshops weisen markante Parallelen zu Endres & Weiblers Beobachtungen und Feststellungen auf. Deren empirisch begründete Gestaltungsempfehlungen (2019, S. 29 ff.) für die Kultivierung von Plural Leadership mittels unterstützter Lerndialoge sowie kollektiver Identitätsbildung und Sinnstiftung erscheinen uns wesensverwandt mit unserem Vorgehen des gemeinsamen Vorantastens in der Führungsentwicklung. Endres & Weibler beschreiben außerdem die Notwendigkeit von „Humble Leadership“ bzw. einer Haltung der Bescheidenheit auf Ebene Top-Management als Gelingensfaktor für Entwicklungsprozesse hin zu Shared Leadership. „Führende dieser Kategorie sind unvoreingenommen sowie lernbereit. Sie regen dies auch auf Teamebene an, etwa indem Raum für gemeinsame Erfahrungen geschaffen wird. Sie verzichten darauf, allzu schnell auf Lösungen zu pochen oder diese selbst zur Verfügung zu stellen“ (2019, S. 37). Der Führungsstil der Bereichsleiterin und des Bereichsfachverantwortlichen wurde in den Rückmeldungen ähnlich beschrieben und es war beiden von Beginn der Führungsentwicklungsreise an wichtig gewesen, mit allen Beteiligten gemeinsam in einen Lernprozess einzusteigen und sich dadurch herausfordern zu lassen, anstatt einen solchen Prozess nur anzuordnen und von einer übergeordneten Position aus zu verfolgen. Dazu gehörte ein zweifacher Vertrauensvorschuss: erstens in den Kreis der Führungspersonen, denn „Führung auf Augenhöhe“ kann nicht einseitig umgesetzt werden, sondern ist abhängig von entsprechender Mitgestaltung durch ihre Adressaten, und zweitens in Richtung der Prozessbegleiterin. Über die Jahre hat sich in beiderlei Hinsicht eine vertrauensvolle und sinnorientierte Zusammenarbeit etabliert, auf deren Basis zunehmend kreativere und mutigere Entwicklungsschritte möglich wurden. Diese nahmen ihre Anfänge in „Generativen Dialogen“ im kleinen Kreis des Prozessentwicklungstrios und wurden dann im Workshopkontext der Intelligenz des Kollektivs übergeben. Dort wurde Führung als „gemeinschaftlicher Einflussprozess“ im Sinne von Endres & Weibler (2019, S. 42) auf Basis von gemeinsamen Zielen und Werten sowie geteilter Verantwortung ko-gestaltet.

Endres & Weibler betonen, dass Führung kein „Ding“ ist, „über das eine ‚Führungskraft‘ frei ‚verfügen‘ kann, sondern ein beziehungsbezogener Prozess“ (ebd.). Das gleiche gilt für Sinn. Sinn kann nicht willkürlich definiert und Sinnempfinden nicht hergestellt, angeordnet oder eingeimpft werden. Sinnbildung entsteht nicht durch Akte der Sinnstiftung seitens heroischer Führungskräfte oder charismatischer Vordenker, sondern es handelt sich dabei nach Kruse um „eine Wir-Aufgabe, bei der man sich auf die Kreativität des Augenblicks und auf Iteration verlassen muss“ (2015, S. 76). Sinn ist also als ein emergentes Phänomen zu verstehen, das sich der dauerhaften Festschreibung oder abstrakten Kodifizierung widersetzt und vielmehr immer wieder neu aus konkreten Situationen entsteht. In den Worten Frankls: Sinn kann weder „gegeben“ noch „erzeugt“ werden, er muss „gefunden“ werden (Frankl 1983, S. 28). Bei der „Sinn-Wahrnehmung handelt es sich um die Entdeckung einer Möglichkeit vor dem Hintergrund der Wirklichkeit. Und diese Möglichkeit ist jeweils einmalig“ (ebd.).

Frankl beschrieb drei „Hauptstraßen“ zum Sinn, die Jung (2020a, 2020b) im Kontext von Arbeits- und Führungsbeziehungen als Möglichkeiten des Werteerlebens und der Werterealisierung fasst: *Schöpferische Werte* werden im Rahmen des Hervorbringens einer „materiellen oder immateriellen Leistung verwirklicht“ (2020b, S. 181). *Erlebniswerte* werden realisiert, wenn das Arbeitsgeschehen, das Miteinander und das hingewendete Beteiligtsein das persönliche Werteempfinden berühren (ebd.). *Einstellungswerte* schließlich drücken sich „durch persönliche Haltungen in schwierigen, nicht oder jedenfalls nicht kurzfristig veränderbaren Situationen“ (2020a, S. 50) aus. „Einer zunächst bedrückenden Situation ein ‚Trotzdem‘ entgegenzustellen, ist immer eine der geistigen Person innewohnende Sinnmöglichkeit“ (ebd.). Die Führungsworkshops im Bereich Medizin boten unserer Einschätzung nach Möglichkeiten zur Sinnentdeckung und -verwirklichung auf allen drei Ebenen. Mit Hinblick auf das Thema Resilienz und die Bewältigung der Pandemiesituation erscheint uns die Kategorie der Einstellungswerte (ausgedrückt in der Haltung gegenüber Ereignissen und Zuständen, auf die kein Einfluss besteht) von besonderer Bedeutung. In der wiederholten, gemeinschaftlichen Auseinandersetzung mit widrigen Umständen und überfordernden Konstellationen hat sich über die Jahre, so unsere Hypothese, eine Art kollektive „Trotzmacht“ als Kraftquelle entwickelt.

Während die Führungsworkshops Räume für das Entdecken und die Entfaltung von Sinn geboten haben, waren sie jedoch nie daraufhin angelegt, einen bestimmten Sinn herzustellen oder top-down zu vermitteln. Es ging gerade nicht um das Einschwören auf einen organisationalen Purpose, noch um das Aufgehen in einem unkritischen Kollektivismus. Stattdessen wurden neben der gemeinsamen Basis und den geteilten Zielen immer auch die Diversität innerhalb der Gruppe und die Unterschiede in den Rollen, Erfahrungen und Sichtweisen in den Blick genommen mit dem Ziel, eine Verbundenheit in Vielfalt zu etablieren. Das Thematisieren von individuellen Wertvorstellungen, auch in ihrer möglichen Abweichung von offiziellen Verlautbarungen der Organisation, kann nach Jung „eine sinnorientierte Arbeitskultur befördern, ja gegebenenfalls erst die Möglichkeiten zur Werterealisierung am Arbeitsplatz bewusst machen“ (2020b, S. 184). Borck warnt Führungspersonen vor der Falle, sich als Sinnstifter zu definieren und dabei den Eigen-Sinn der Mitarbeitenden zu missachten, denn diese entscheiden eigenständig darüber, ob sie einen Fremd-Sinn annehmen oder nicht. Er ersetzt den Begriff Sinnstiftung mit Sinnkoppelung und spricht von sinngekoppelten und -entkoppelten Mitarbeitenden (zitiert in Jumpertz 2015, S. 75). Wir stimmen insofern zu, dass es nicht um die Frage geht, wie Management top-down Sinn stiften oder vermitteln kann, sondern vielmehr darum, wie Sinnprozesse auf Augenhöhe gestaltet und Sinngemeinschaften gepflegt werden können. Hinterfragen möchten wir jedoch die strikte Trennung von Eigen- und Fremd-Sinn.

Auch wenn das persönliche Sinnempfinden ein individuelles ist bzw. jeder Mensch seine einzigartigen Sinnmöglichkeiten zu finden hat (Frankl 1981), so geschieht dies jedoch nicht in einem subjektivistischen Vakuum. Zwar können Menschen einander keinen Sinn überstülpen oder entscheiden, was für andere sinnvoll wäre. Daraus folgt aber nicht, „wir selbst bestimmen den Sinn […], was der einzelne für sinnvoll hält, wird einfach als Sinn definiert“ (Berschneider 2003, S. 44). Sinn ist vielmehr ein „transsubjektives“ (ebd.), soziokulturelles Phänomen. „Der Sinn des Sinns ist es eben, wie es Jürgen Habermas formuliert hat, dass er geteilter Sinn ist […]. Daher ist Privatsinn letztlich Unsinn bzw. können Individuen für sich allein nicht über den Sinn verfügen, weder den Sinn ihres Tuns noch ihres Erlebens“ (Reichenbach 2018, S. 106). Ein Selbst kann man nach Reichenbach nur mit und durch andere sein, und auch Sinn erfahren wir nicht selbstbestimmt oder selbstkreiert, sondern als Mitglieder von Sprach- und Wertegemeinschaften sowie bezogen auf geteilte Welten. Sinn muss zudem immer wieder neu gestärkt werden durch Kommunikation und Kultur, also durch überindividuelle Praktiken. Sinnempfinden setzt damit die Fähigkeit voraus, sich in einem sozialen Rahmen zu sehen, wobei die „Intersubjektivität vorranging ist vor der Subjektivität“.^4^

Bei der Sinnverwirklichung geht es auch nicht um Selbstverwirklichung, diese ist mehr als ein Nebeneffekt zu betrachten. Es geht darum, sich in den Dienst von etwas Selbsttranszendentem zu stellen (Berschneider 2003). Positives Wirken in der Welt ist zudem selten ein individuelles Phänomen, sondern Selbstwirksamkeit kommt dann am besten zur Geltung, wenn sie sich mit der anderer Akteure verbindet. Künkel verweist in diesem Zusammenhang auf eine vielzitierte Aussage der Anthropologin Margaret Mead: „Zweifle nie daran, dass eine kleine Gruppe von entschiedenen Menschen die Welt verändern kann, … tatsächlich ist dies die einzige Art und Weise, in der die Welt jemals verändert wurde“ (2016, S. 152). Die eigene Selbstwirksamkeit zu multiplizieren durch die Verbindung mit einem an Sinnhaftigkeit und zukunftsfähigem Handeln ausgerichteten Kollektiv sieht sie als eine wesentliche Komponente sinnorientierter Führung.

Die Sinnforscherin Schnell betont ebenfalls die Bedeutung des Kriteriums Zugehörigkeit: „Sinnerleben verlangt, dass wir nicht das Gefühl haben, dass wir allein auf der Welt sind, vereinzelt und isoliert, sondern dass wir irgendwo dazugehören“ (Schnell 2014). Auch wenn der vorherrschende Zeitgeist Selbstoptimierung und Eigennutz in den Vordergrund stellt, bestätigt ihre Forschung, dass es Menschen besser geht, wenn sie etwas für andere tun, anstatt für sich selbst. Weiterhin nennt sie „Generativität“ als stärksten Sinnprädiktor; gemeint ist das Leisten von Beiträgen für ein größeres Ganzes. Sinn entwickelt sich im „aktiven Weltbezug“ (Schnell 2016, S. 8), in Taten mehr als in Worten. Er entsteht dann, wenn Werte aktiv umgesetzt und gelebt werden können.

Jedoch stellt Schnell (2014) ein zunehmendes Schwinden der subjektiven Wahrnehmung eines größeren Ganzen fest parallel zu einem Verlust von Zugehörigkeits- und Zusammenhaltsempfinden im gesellschaftlichen Raum. Womöglich ist es ein solches Vakuum, vor dem die derzeitige Popularität organisationaler Bestrebungen der Sinn- und Identitätsstiftung u. a. verstanden werden muss – als Substitut für verlorengegangenen Gemeinsinn. Es scheint aber fraglich, ob eine symbolisch-theoretische Auseinandersetzung mit Sinnfragen jenseits realer Beziehungsnetzwerke Sinnquellen wie Zugehörigkeit, gemeinschaftlich gelebte Werte und eine im konkreten Tun praktizierte kollektive Bewältigung von Sinnanfragen ersetzen kann. Angesichts des Schwunds an Zugehörigkeitsräumen und geteilten, identitätsstiftenden Grundüberzeugungen besteht vielmehr die Gefahr, dass „Management by Sinnvermittlung“ in Richtung einer Karikatur von Sinn abdriftet oder auch in Richtung Manipulation von Sinnbedürfnissen, womit eine neue Stufe der Ausbeutung erreicht wäre (vgl. Kruse 2015).

Vor diesem Hintergrund erscheint es uns zentral, Sinninhalte nicht losgelöst von den tatsächlichen Gemeinschaften zu denken, in welchen sie entstehen und gelebt werden. Der Begriff des „Sozialkapitals“ verweist auf Netzwerke zwischenmenschlicher Beziehungen sowie geteilter Überzeugungen, Werte und Regeln als systemische Voraussetzungen für gelingende Kooperation (Badura et al. 2013). „Menschen brauchen Menschen, um sich gegenseitig zu motivieren, um ihre kreativen Kräfte freizusetzen, um ihr Bedürfnis nach Aufmerksamkeit und Anerkennung zu befriedigen und um Ziele zu erreichen und Herausforderungen zu bewältigen, die sie allein nicht erreichen oder bewältigen können“ (2013, S. 26). Hierzu passt die in unseren Interviews und in vielen informellen Gesprächen immer wieder erwähnte Figur der „Kraft des großen Kreises“. Das Vertrauen in diese Kraft und die emotionale Verbindung zu diesem Kreis scheinen uns wesentliche Einflussfaktoren auf das Sinnerleben gerade in schwierigen Zeiten gewesen zu sein.

Sinn ist also nicht willkürlich top-down oder subjektivistisch autonom definierbar, sondern entsteht in unserem Beispiel, so unsere Schlussfolgerung, in fortdauernden Beziehungen und im kontinuierlichen gemeinsamen Ringen um Orientierung und Selbstwirksamkeit angesichts permanenten Wandels und wachsender Unsicherheit. Die Führungsworkshops unterstützten den reflexiven Sinngewinn aus gemachten Erfahrungen sowie das vorwärtsgerichtete Gestalten von geteilten aktuellen und zukünftigen Sinnzusammenhängen. Gleichzeitig stellten sie Investitionen in das Sozialkapital des Bereichs dar, indem sie die Qualität der internen Vernetzung sowie den Vorrat an gemeinsamen Werten und Überzeugungen erhöhten, emotionale Bindungen, Kohäsion und Kohärenz förderten, Kooperation erleichterten und Energie für gemeinschaftliche Zielverfolgung lieferten (vgl. Badura et al. 2013, S. 10).

## Abschließende Reflexion und Ausblick

Der konstruktive Umgang mit instabilen Kontexten, steigender Veränderungsdynamik, Ambiguität und vielfältigen Spannungsfeldern ist heute zur zentralen Führungsaufgabe avanciert und kann nicht individuell geleistet werden, sondern erfordert die Kompetenz zum Zusammenwirken mit anderen. Künkel spricht von einer „Aufforderung zum Resilienz-Training“ zur Vorbereitung auf das, „was in Zukunft an der Tagesordnung sein wird – komplexe Veränderungsprozesse gemeinsam mit unterschiedlichen Akteuren in unsicherem Umfeld angehen und dabei agil und schnell dazulernen“ (2016, S. 153). Führungsverantwortliche sind gefordert, hierfür die geeigneten Rahmenbedingungen zu gestalten und Teams und Einzelne zu ermächtigen, Handlungsspielräume auszuschöpfen und situativ immer wieder neue Lösungen zu finden. Im Kontext unseres Fallbeispiels ist es gelungen, eine bejahende Haltung zum permanenten „Change“ sowie eine selbstorganisierte Anpassungsfähigkeit unter Unsicherheitsbedingungen zu fördern. Das Führungskollektiv der Pflege im Bereich Medizin des USB hat sich dahin entwickelt, Herausforderungen gemeinsam anzugehen und auch in unsicheren Phasen auf Chancen zu fokussieren. Man könnte von einer resilienzzentrierten Führung sprechen, die Instabilität und Ungewissheit nicht zu vermeiden sucht, sondern als Gestaltungsaufgabe annimmt (vgl. Wüthrich 2016; Kruse 2004). Gemeinsam hat man sich „das Chaos zum Freund gemacht“ und als Quelle für Kreativität und Entwicklung genutzt, womit es zum Schutz vor „zu großem Chaos“ werden konnte (vgl. Künkel 2016, S. 79 f.) – z. B. in der Pandemiesituation.

Die regelmäßigen Führungsworkshops boten Raum dafür, Metaperspektiven einzunehmen und in der gemeinsamen Selbstbeobachtung und Selbstreflexion die individuellen und kollektiven Wahrnehmungsfähigkeiten zu erweitern sowie dialogisch gewonnene Erkenntnisse in neue Handlungsmöglichkeiten zu übersetzen. Im Kontext verschiedener Experimente (z. B. szenisches Arbeiten, Simulationen, Song-Aufnahme) waren die Teilnehmenden immer wieder gefordert, ihre Komfortzone zu verlassen und Irritationen auszuhalten. Dies förderte die Freisetzung spontaner Kreativität, das Erproben ungewohnter Denk- und Herangehensweisen und das Herausbilden neuer Anschauungen und Praktiken. Im gemeinsamen Erkunden und Ausprobieren entstand eine Lern- und Entwicklungsgemeinschaft, durch den Bezug zur geteilten Führungsverantwortung zudem eine Sinngemeinschaft.

Weiterhin stellten die Führungsworkshops einen Rahmen für die Verankerung und kontinuierliche Weiterentwicklung einer Vision von gemeinschaftlicher Führung und einer entsprechenden Werthaltung dar. Man setzte sich mit einer Palette von diesbezüglichen Instrumenten und Denkanstößen auseinander und begab sich immer wieder in Dialoge und Aushandlungen darüber, wie diese Vision im Alltag gemeinsam gelebt werden kann. In den Interviews wurde durchgängig ein Gefühl der Verbundenheit als gemeinsames Fundament beschrieben – trotz der Tatsache, dass man im Alltag relativ verstreut ist. Angesichts der COVID-19-Pandemie, aber auch im Kontext zahlreicher weniger dramatischer Krisen- und Drucksituationen, trat das entsprechende Potenzial auf eindrückliche Weise zum Vorschein.

Neben den erfreulichen Schlüssen, welche sich aus der retrospektiven Analyse der Führungsentwicklung ableiten lassen, sind aber auch Grenzen festzustellen. So operiert die Pflege im Klinikkontext nicht unabhängig von anderen Professionen. Schon früh in der Führungsentwicklungsreise wurde die Ärzteschaft als zentraler Partner zum Thema – wobei jedoch das Miteinander im Alltag häufig als nicht gerade partnerschaftlich beschrieben wurde. Versuche in Richtung eines Einbezugs der ärztlichen Kolleginnen und Kollegen in die Führungsentwicklung waren nur punktuell erfolgreich und zerliefen sich mehrheitlich an den parallelen Hierarchien und Prioritäten der beiden Berufsgruppen. Hier liegt noch enormes, bisher unerschlossenes Potenzial, welches aber nicht einseitig durch die Pflege mobilisiert werden kann. Die anstehende Reorganisation des USB könnte in dieser Hinsicht neue Chancen eröffnen.

In den Interviews fragten wir auch nach Verbesserungsideen und Vorschlägen für alternative Vorgehensweisen. Mehrere Personen regten an, dass kürzere Treffen im kleineren Kreis (z. B. zum Zweck von kollegialer Fallberatung) eine sinnvolle Ergänzung zu den Workshops darstellen würden. Tatsächlich hatten wir solche Formate einige Male angeboten bzw. angeregt. Es waren daraus aber keine selbstorganisierten Fortsetzungen entstanden. Obwohl in anderer Hinsicht Impulse weitergetragen wurden – z. B. haben vielfach Abteilungsführungsteams Anregungen und Experimente aus den Workshops in eigenen Veranstaltungen mit ihren Teams wiederholt – so gab es bisher in punkto abteilungsübergreifender Führungsentwicklung keine eigenständigen Initiativen. Bei einem Fortbestand des Bereichs würden wir im Sinne von Shared Leadership ein zentrales nächstes Entwicklungsziel darin sehen, dass nicht nur die gemeinschaftliche Alltagsbewältigung, sondern auch die zukunftsorientierte gemeinsame Weiterentwicklung zumindest teilweise in Selbststeuerung initiiert und verfolgt würde.

In der Vorbereitung auf die geplante Fusion mit einem anderen Klinikum haben wir uns 2018 angesichts all der Veränderungsziele auch mit Bewahrungszielen bzw. dem Erhaltenswerten an der bestehenden Kultur im Bereich beschäftigt. Sinnbildlich gesprochen ging es darum, welches „Tafelsilber“ in den neuen gemeinsamen Haushalt eingebracht werden sollte. Ähnliche Überlegungen ergeben sich aktuell aus der gerade angekündigten Reorganisation des USB, mit der die klassische Trennung zwischen den Bereichen Medizin und Chirurgie aufgehoben wird. Damit einher geht die Auflösung des Bereichs Medizin und die Verteilung der Abteilungen auf neu entstehende Departemente, wo sie mit Abteilungen aus der Chirurgie und den Spezialkliniken zusammengeführt werden. Ob dies einer „Kulturrevolution“ (Hoffmann 2020) gleichkommt, sei hier dahingestellt. Sicherlich werden aber unterschiedliche Kulturen und Führungsverständnisse aufeinandertreffen und neue Aushandlungsprozesse vonnöten sein.

Mit Blick auf das im Bereich Pflege Medizin Erreichte und Erhaltenswerte bietet diese Situation Risiken und Chancen. Badura et al. (2013) verweisen auf das Sozialkapital als Teil des immateriellen Vermögens einer Organisation und einen bisher stark unterschätzten Erfolgsfaktor. Die Reorganisation könnte zu einem Verlust oder einer Minderung von Sozialkapital führen, aber alternativ auch zu dessen Erweiterung, wenn es gelingt, den Zusammenhalt weiter zu pflegen und gemeinsam mit neuen Partnern zur Kernaufgabe im Veränderungsprozess zu machen. Ein letzter Workshop in der „alten“ Konstellation, der im Herbst hoffentlich auch unter Corona-Bedingungen wird stattfinden können, soll hierfür zur Vorbereitung dienen, wichtige Weichen stellen und Vertrauens- und Stabilitätsanker setzen für die konstruktive Bewältigung des Wandels. Große Veränderungen werfen fast zwangsläufig Fragen der Sinnhaftigkeit auf. Diesen wollen wir uns – wieder einmal – gemeinsam stellen.

## Footnotes

1 Zu diesem Bereich gehörten bis 2019 insgesamt 17 Abteilungen: acht Bettenstationen, sieben Ambulatorien, das Notfallzentrum und die Medizinische Intensivstation.

2 Die Berufsbildung spielt nicht nur für die Nachwuchsentwicklung und somit die Zukunft der Pflege eine zentrale Rolle, sondern auch im Stationsalltag leisten die Bildungsverantwortlichen essentielle Beiträge auf Führungsebene, z. B. bei der Einführung von neuen Mitarbeitenden, bei der Umsetzung von Leitlinien und Neuerungen, im Kontext der Zusammenarbeit verschiedener „Skills & Grades“ (unterschiedlicher Fähigkeitsstufen und Berufsabschlüsse) sowie bei Werteklärungen.

3 Vielversprechend erscheinen uns u. a. die Complexity Leadership Theory (z. B. Uhl-Bien et al. 2007) mit ihrem Fokus auf emergente Dynamiken und kollektive Einfluss- und Lösungsfindungsprozesse sowie jüngste Anknüpfungen an das Resonanz-Konzept des Soziologen Hartmut Rosa mit Bezug zu Führung (Eibisch 2019) bzw. zu organisationalen Veränderungsprozessen (Pfleghar und Hott 2020).

4 R. Reichenbach, persönliche Kommunikation (30.06.2020).
